# Editorial: Reviews in non-neuronal cells 2024 & 2025

**DOI:** 10.3389/fncel.2026.1825487

**Published:** 2026-03-27

**Authors:** Qingchao Qiu, Bo Hu

**Affiliations:** 1 Michael E. DeBakey Veterans Affairs Medical Center, Department of Veterans Affairs, Houston, TX, United States; 2 Department of Medicine, Baylor College of Medicine, Houston, TX, United States; 3 Department of Neurology, Houston Methodist Research Institute, Houston, TX, United States; 4 Department of Neurology, Weill Cornell Medical College, New York, NY, United States

**Keywords:** astrocytes, endothelial cells, glia cells, macrophages, microglia cells, Müller cells, neurovascular unit, non-neuronal cells

## Introduction

The Non-Neuronal Cells section in *Frontiers in Cellular Neuroscience* is delighted to present this Reviews in series highlighting the Roles and Mechanisms of Non-Neuronal Cells in Health and Disease. Although the nervous system has traditionally been framed as a primarily neuronal network (Furness, 2000; Yuste, 2015), it is now clear that non-neuronal cells are active and indispensable regulators of nervous system development and function. Across the central and peripheral nervous systems, glial, immune, and vascular-associated cell types shape circuit formation and plasticity, regulate vascular dynamics and barrier integrity, support metabolic homeostasis, coordinate immune surveillance, and drive tissue repair programs through inflammatory signaling, trophic support, and direct cell–cell interactions (Sjoberg et al., 1988; Vahsen et al., 2021; Cathomas et al., 2022). Astrocytes, Schwann cells, oligodendrocyte lineage cells, microglia, border-associated macrophages, neurovascular unit components such as endothelial cells, pericytes, and perivascular macrophages, as well as peripheral immune populations, collectively influence the neural microenvironment through contact-dependent mechanisms and soluble mediators (Sano et al., 2007; Sa-Pereira et al., 2012; Domingues et al., 2016; Hu et al., 2018; Cathomas et al., 2022; Sun and Jiang, 2024). A key example is gliotransmission, whereby glial cells release signaling molecules such as glutamate, ATP, D-serine, adenosine, GABA, TNF-α, and BDNF to influence synaptic activity and network state (Araque et al., 2014; Sancho et al., 2021; Yu et al., 2024). Through these interactions, they coordinate neurovascular coupling, neuroimmune communication, gliotransmission, extracellular and ionic homeostasis, and waste clearance, thereby influencing neurodevelopment, neuroprotection, and the onset and progression of neurodegenerative diseases (Benarroch, 2005; Shaham, 2005; Suzumura, 2013; Puentes et al., 2016; Vahsen et al., 2021; Taylor and Monje, 2023).

**Figure 1:**
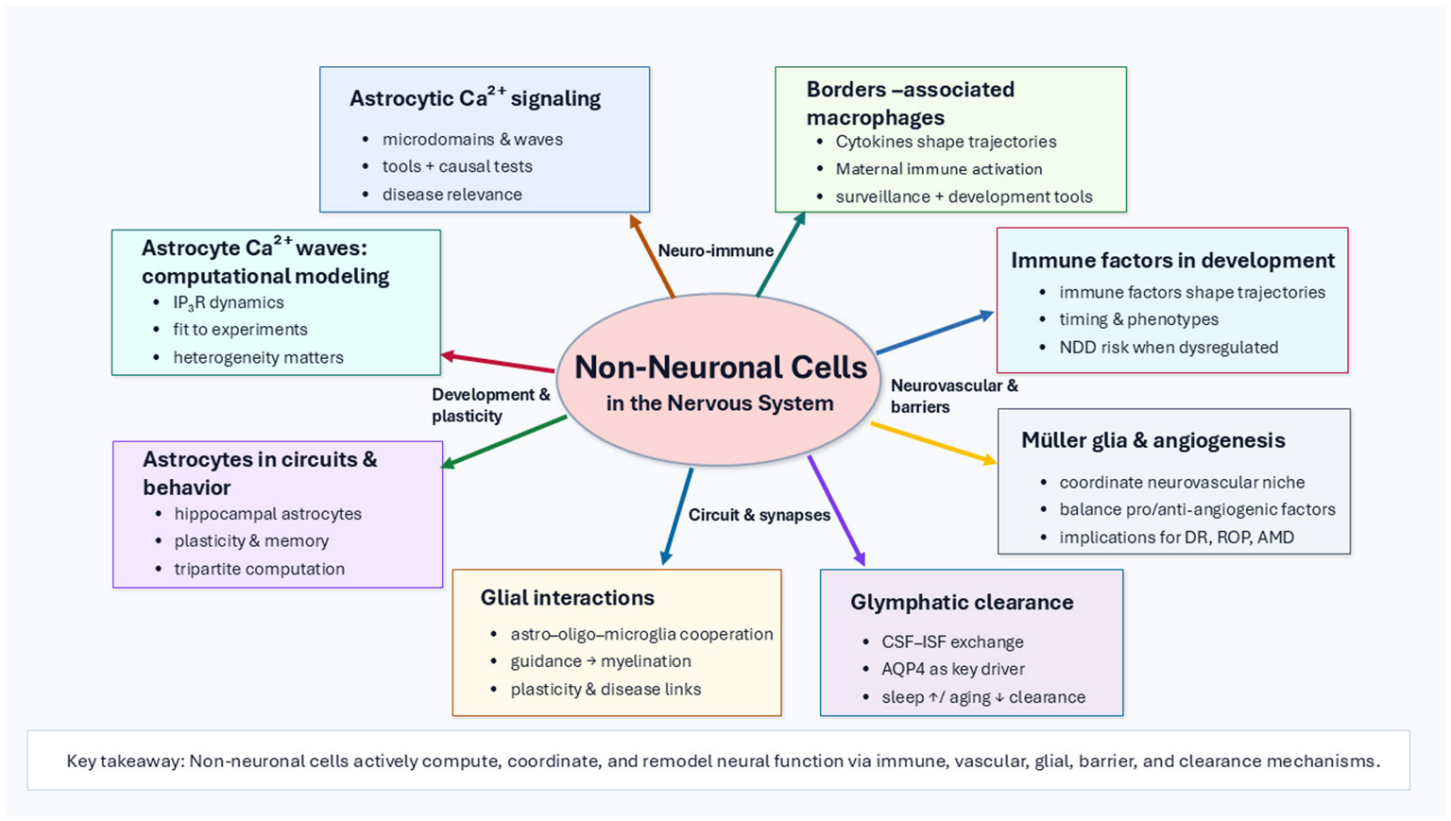
Summary of the eight articles in the Research Topic “*Reviews in non-neuronal cells 2024 & 2025*”

This Research Topic brings together eight review articles spanning astrocyte signaling and computation, developmental glial coordination of long-range connectivity, brain-wide clearance physiology, retina gliovascular biology, and the expanding landscape of nervous system immune interfaces Figure 1. Collectively, these contributions underscore a unifying principle: non-neuronal cells do not merely respond to neural activity—they help define the neural microenvironment that enables or constrains neuronal function and vulnerability. Below, we summarize the key themes and advances across the Research Topic and highlight emerging priorities for the field.

## Astrocytic calcium signaling as a multi-scale language of glial function

Astrocytic calcium signaling as a foundational language for astrocyte–neuron communication (Nowacka et al., 2025). Chen, Ye et al. provide a broad synthesis of astrocytic Ca^2+^ signaling sources, classifications, detection approaches, and functional significance, emphasizing how Ca^2+^ signals extend beyond the soma into fine processes and endfeet to coordinate synaptic plasticity, neurotransmitter regulation, and homeostatic control. Importantly, the review frames Ca^2+^ signaling as a disease-relevant hub—linking network activity to blood flow and metabolism—and calls for cross-modal, multi-scale measurements and causal tests to decode signal “meaning” across contexts.

Extending this mechanistic perspective, Musotto et al. address a pressing need in glial research: quantitative models that faithfully capture calcium wave dynamics. They compare established computational formulations (e.g., Goldbeter, De Young–Keizer, Atri, Li-Rinzel, De Pittà) and emphasize that matching real astrocyte behavior requires tight integration of modeling with experimental measurements. Their analysis illustrates how discrepancies between theoretical oscillatory patterns and observed Ca^2+^ time courses can reveal missing biological mechanisms, guide parameter optimization, and refine model structure—an approach essential for moving from “astrocytes can signal” to “astrocytes compute.”

## Astrocytes as circuit partners: from tripartite synapses to behavior

Squires and Park extend astrocyte Ca^2+^ signaling to circuit-level outcomes, presenting astrocytes as integrators and modulators of hippocampal computation. Centered on the concept of tripartite synapses, the review describes how astrocytes can tune synaptic function within their territorial domains while also coordinating activity across larger networks, thereby shaping oscillations, plasticity, and behavioral readouts related to learning and memory. The authors further highlight state dependence—particularly sleep and wakefulness—where astrocytic Ca^2+^ dynamics vary systematically and can influence sleep architecture and hippocampal rhythms. By framing astrocytes as active elements in circuit computation (rather than passive buffers), this review helps connect cellular signaling mechanisms to emergent properties of behavior and neurodegeneration.

## Glial collaboration in wiring the brain: corpus callosum development and plasticity

Czyrska et al. broaden the lens from local circuits to long-range connectivity by reviewing how astrocytes, oligodendrocytes, and microglia collaborate during corpus callosum formation and remodeling. The review emphasizes astrocytic “guidepost” roles at the midline—structurally and chemically shaping axonal navigation—while oligodendrocyte lineage cells enable maturation and activity-dependent myelination that supports efficient conduction and plasticity. Microglia add a complementary layer of developmental quality control through pruning and refinement mechanisms, integrating immune-like functions into circuit assembly. Beyond development, the authors highlight translational directions, including multimodal single-cell approaches and human-relevant models (iPSC-derived systems, organoids/assembloids) to connect glial mechanisms to congenital malformations and neuropsychiatric outcomes. This review exemplifies a conceptual shift: neural connectivity is not solely an axonal “engineering” problem, but a multicellular choreography in which glia provide scaffolding, guidance, metabolic support, and immune-mediated refinement.

## Brain-wide homeostasis: astrocytes at the center of glymphatic clearance

The glymphatic system review by Chen, Wang et al. provides a timely synthesis of cerebrospinal fluid–interstitial fluid (CSF–ISF) exchange and brain waste clearance, emphasizing the central role of astrocytic endfeet and aquaporin-4 (AQP4) in perivascular fluid movement. The review highlights that glymphatic function is enhanced during sleep, declines with aging, and is implicated in neurodegenerative and vascular conditions. A particularly actionable focus is the evidence linking AQP4 impairment or depolarization to reduced amyloid clearance and greater amyloid accumulation—supporting the broader concept that astrocyte polarity and endfoot organization are not structural details but determinants of proteostasis and disease trajectory.

## Müller glia as angiogenic gatekeepers

Non-neuronal regulation of vascular biology is further developed in Medina-Arellano et al.' review of Müller glia as critical regulators of retinal angiogenesis in health and in vasoproliferative retinopathies. The authors highlight Müller cell secretome control over pro- and anti-angiogenic signaling and argue that future therapies may benefit from shifting the retinal microenvironment toward anti-angiogenic and neuroprotective states by modulating Müller glial cytokine/growth factor release—potentially earlier than overt vascular pathology. This review reinforces a theme across the Research Topic: barriers and vascular interfaces are core arenas where non-neuronal cells set the rules of tissue resilience.

## Immune systems and nerve development: cytokines, microglia, and border immunity

Two reviews focus on immune mechanisms in neurodevelopment, emphasizing that immune signaling is not merely reactive but instructive during brain formation. Wang et al. review the roles of cytokines, microglia, astrocytes, and border immune cells in shaping neurogenesis, synaptic pruning, and inflammatory programming, with particular attention to how dysregulated immune signaling contributes to neurodevelopmental disorders. The review synthesizes evidence from maternal immune activation models and highlights cytokine-mediated pathways (including IL-6/IL-17-associated mechanisms) as plausible bridges between prenatal inflammation and long-term circuit alterations. Otero and Antonson focus on border-associated macrophages (BAMs)—tissue-resident macrophages positioned at meninges, choroid plexus, and perivascular spaces—arguing that their strategic location enables immune surveillance, vascular modeling, debris clearance, and influences on CSF dynamics during development. The review details yolk-sac-derived origins and early colonization timelines, while also acknowledging important open questions regarding lineage relationships and region-specific diversification.

## Concluding remarks

These eight reviews reinforce a modern view of the nervous system as an ecosystem in which non-neuronal cells define context—setting metabolic tone, tuning synaptic and network behavior, organizing axon guidance and myelination, regulating vascular and barrier function, and shaping immune signaling in space and time. Across topics, a clear methodological trajectory emerges: single-cell and spatial multi-omics to define states and lineages; quantitative imaging (often at subcellular resolution) to decode signaling dynamics; computational modeling to connect mechanisms to observed patterns; and cell-type-specific perturbations to establish causality.

As the field advances, key challenges will be to harmonize measurements across scales (molecules → microdomains → circuits → behavior), map how compartmental interfaces (perivascular, meningeal, choroid plexus, retinal barriers) reshape signaling logic, and translate mechanistic insights into therapies that target non-neuronal pathways without disrupting essential homeostatic functions (Ben Haim and Rowitch, 2017; Gao et al., 2023; Verkhratsky et al., 2023; Tsitsou-Kampeli et al., 2024; Vishnumukkala et al., 2025). By spotlighting diverse non-neuronal cell types and processes, this Research Topic aims to catalyze cross-disciplinary approaches and accelerate discovery of glia- and immune-informed strategies for understanding—and ultimately treating—neurological disease.
